# Are work return and leaves of absence predictable after an unstable pelvic ring injury?

**DOI:** 10.1007/s10195-015-0379-2

**Published:** 2015-09-28

**Authors:** Alessandro Aprato, Alexander Joeris, Ferdinando Tosto, Vasiliki Kalampoki, Elke Rometsch, Marco Favuto, Alessandro Stucchi, Matheus Azi, Alessandro Massè

**Affiliations:** Medical School, University of Turin, Turin, Italy; Clinical Investigation and Documentation (C.I.D.) Department, AO Foundation, Dübendorf, Switzerland; Manoel Victorino Hospital, Salvador, Brazil

**Keywords:** Pelvic fracture, Pelvic ring injuries, Morbidity, Productivity loss, Leave of absence

## Abstract

**Background:**

Resuming work after surgical treatment of an unstable pelvic ring injury is often impeded because of residual disability. The aim of this study was to test which factors influence return to work, ability to return to the same job function as before the injury, leaves of absence, and incapacitation after sustaining a pelvic fracture.

**Materials and methods:**

We performed a retrospective study on patients with surgically treated pelvic fractures. Medical records were reviewed to document patients’ demographic data, the extent of follow-up care, diagnosis of the injury (according to the Tile system of classification), type of surgical treatment, injury severity, and the time from trauma to definitive surgery. We also recorded the classification of patients’ physical status according to the American Society of Anesthesiologists (ASA) and details about admission to the intensive care unit (ICU). Patients were interviewed to note the number of days before returning to work and their ability to maintain their previously held jobs.

**Results:**

Fifty patients were included in the study, and their mean age was 46.3 ± 12.6 years. The median time to return to work was 195 days. Twelve patients (24 %) lost their jobs and 17 (34 %) resumed their previous job with a change of tasks. ICU admission and time from trauma to definitive surgery were negatively correlated with return to the previously held job. Returning to the same job tasks was not associated with any of the factors investigated. Polytrauma, ICU admission, and time from trauma to definitive surgery were associated with longer leaves of absence.

**Conclusions:**

Work reintegration after pelvic ring injuries is a major issue for patients and health care systems: 58 % of patients were not able to return to or lost their job. Factors correlated with leaves of absence were injury severity, delayed definitive fixation, and ICU admission.

**Level of evidence:**

IV (case series).

## Introduction

Morbidity and complications are frequent after an unstable pelvic injury [1]. Patients usually undergo a long rehabilitation and injury often creates a chronic disabling condition, which frequently requires long-lasting analgesic therapies and impairs the ability to work [2]. The latter generates a huge societal impact through loss of employment or the need for professional retraining. Many studies [3–18] have shown low rates of job reintegration after a pelvic fracture but, to our knowledge, the association between days of work absence and pre-trauma health status, the severity of injury, and job characteristics has not yet been investigated.

We performed a retrospective study on patients with pelvic ring injuries treated surgically in our referral center. The main aim of this study was to evaluate the type of fracture, pre-trauma health status, time from trauma to definitive surgery, severity of injury, and job characteristics, and their influence on work resumption, the ability to maintain the previously held job, and leaves of absence.

## Materials and methods

Between 2010 and 2012, we surgically treated 93 pelvic ring injuries in our referral center. All fractures were operated upon by at least two surgeons of our pelvic surgery team, which consists of three specialized surgeons. The study was approved by the local ethics committee and was conducted in accordance with the ethical standards laid down in the 1964 Declaration of Helsinki and its later amendments. Hospital charts were retrospectively reviewed after patients had given informed consent to the use of their data.

Patients were considered ineligible if they did not work before the trauma (e.g., students and retirees), if they had been operated on less than 9 months previously so that no follow-up of 9 months or longer was possible, or if no phone contact information was available in the medical records. Data on demographics, diagnoses (according to the Tile system of classification [19]), type of surgical treatment, Injury Severity Score (ISS) [20] on arrival, American Society of Anesthesiologists’ (ASA) physical status classification [21], intensive care unit (ICU) admission, time from trauma to definitive surgery, and follow-up were retrieved from medical records and recorded in a custom database.

Fractures were grouped into B and C types, according to the Tile classification system. The pre-trauma health status was classified as morbid for ASA scores higher than one. Polytrauma was defined as an ISS score of greater than 15 points on hospital admission [22].

Patients were interviewed by phone about their type of work, their ability to return to work, number of days before returning to work, and their ability to return to the same job tasks.

### Statistical methodology

All data were analyzed with standard descriptive statistics. Univariate analysis was performed with regard to (1) readmission to the former job (yes or no) and (2) maintenance of the same job tasks (yes or no). This was done with the chi-squared test or Fisher’s exact test for categorical outcomes and Student’s *t* test or the Mann–Whitney test for continuous outcomes. The Kolmogorov–Smirnov test was used to determine whether data were normally distributed. The relationship between leaves of absence and study characteristics was assessed with univariate linear regression models. Since the values for days off work were skewed, they had to be log-transformed to use them in the regression models. As a consequence, regression coefficients and 95 % confidence intervals were converted into a percent increase in the respective variable using the formula [exp(β)−1] × 100. *P* values lower than 0.05 were considered statistically significant. All analyses were performed using Stata version 12 (Stata Corporation, College Station, TX, USA).

## Results

Of the 88 patients surgically treated for a pelvic fracture in our referral center, 12 patients were lost to follow-up, 15 were excluded because their surgery had taken place less than 9 months before the start of the study, 6 patients were students, and 5 had retired before the trauma occurred. Thus, 50 patients were included in the analysis. Their mean age was 46.3 years (range 18–83) and men represented a higher proportion (64 %). Demographic, clinical, and job-related data are presented in Table 1. Almost 75 % of the study patients suffered a type C fracture. Of the 38 (76 %) patients who returned to their previous job, 20 (54 %) managed to maintain the same job tasks. The median time to return to work was 195 days (range 150–300).

**Table 1:** Demographic, clinical, and job-related characteristics of the study participants

Variables	
Gender	
Female	18 (36.0)
Male	32 (64.0)
Age at surgery (years)	46.3 ± 12.6
Time from trauma to definitive surgery (days)	11 (6; 15)
Follow-up period (months)	38.5 (18; 60)
Type of fracture	
B	12 (24.0)
C	38 (76.0)
Polytrauma (ISS >15)	
No	36 (72.0)
Yes	14 (28.0)
ASA classification score	
0–1	40 (80.0)
2–3	10 (20.0)
ICU admission	
No	18 (36.0)
Yes	32 (64.0)
Sedentary worker	
No	25 (50.0)
Yes	25 (50.0)
Job sector	
Private	40 (80.0)
Public	10 (20.0)
Resumption of the previous job	
No	12 (24.0)
Yes	38 (76.0)
Maintenance of the same job tasks^a^	
No	17 (46.0)
Yes	20 (54.0)
Time to get back to work (days)^a^	195 (150; 300)

Data are presented as *N* (%), mean ± standard deviation, or median (p25; p75)

*ASA* American Society of Anesthesiologists physical status classification; *ICU* intensive care unit

^a^Of the 38 patients who resumed their previous job, information regarding maintenance of the same job tasks was not available for one patient

The analysis of potential predictors for returning (*N* = 38) or not returning (*N* = 12) to the previously held job revealed that job resumption was significantly associated with ICU admission (*p* = 0.036); of the 12 patients who lost their jobs, 11 had been in an ICU (Table 2). There was some evidence that job resumption was also associated with the time elapsing between trauma and definitive surgery; a longer time appeared to have a negative effect on return to work (*p* = 0.047). All remaining characteristics (gender, age at surgery, type of fracture, ISS, pre-trauma health status, sedentary work, and job sector) were not associated with return to work (all *p* values >0.05).

**Table 2:** Demographic, clinical, and job-related characteristics of the study participants according to (a) resumption of the previous job and (b) maintenance of the same job tasks along with univariate association tests

Variables	Resumption of the former job		*P* value	Maintenance of the same job tasks			*P* value
	No (*N* = 12)	Yes (*N* = 38)		No (*N* = 17)	Yes (*N* = 20)		
Gender			0.639^*^				0.717^*^
Female	5 (41.7)	13 (34.2)		5 (29.4)		7 (35.0)	
Male	7 (58.3)	25 (65.8)		12 (70.6)		13 (65.0)	
Age at surgery (years)	46.1 ± 9.8	46.4 ± 13.5	0.937^†^	46.2 ± 12.4		47.5 ± 14.4	0.768^†^
Time from trauma to definitive surgery (days)	14.5 (9; 21.5)	10 (5; 15)	0.047^‡^	12 (8; 15)		7.5 (5; 13)	0.069^‡^
Type of fracture			0.705^§^				0.288^§^
B	2 (16.7)	10 (26.3)		3 (17.6)		7 (35.0)	
C	10 (83.3)	28 (73.7)		14 (82.4)		13 (65.0)	
Polytrauma (ISS >15)			0.718^§^				0.763^*^
No	8 (66.7)	28 (73.7)		12 (70.6)		15 (75.0)	
Yes	4 (33.3)	10 (26.3)		5 (29.4)		5 (25.0)	
ASA classification score			0.686^§^				0.999^§^
0–1	9 (75.0)	31 (81.6)		14 (82.4)		17 (85.0)	
2–3	3 (25.0)	7 (18.4)		3 (17.6)		3 (15.0)	
ICU admission			0.036^§^				0.272^*^
No	1 (8.3)	17 (44.7)		9 (52.9)		7 (35.0)	
Yes	11 (91.7)	21 (55.3)		8 (47.1)		13 (65.0)	
Sedentary worker			0.508^*^				0.072^*^
No	7 (58.3)	18 (47.4)		11 (64.7)		7 (35.0)	
Yes	5 (41.7)	20 (52.6)		6 (35.3)		13 (65.0)	
Job sector			0.416^§^				0.137^§^
Private	11 (91.7)	29 (76.3)		15 (88.2)		13 (65.0)	
Public	1 (8.3)	9 (23.7)		2 (11.8)		7 (35.0)	

Data are presented as *N* (%), mean ± standard deviation, or median (p25; p75)

*ASA* American Society of Anesthesiologists physical status classification; *ICU* intensive care unit

*P* value derived from ^*^Chi-squared test; ^†^ Student’s *t* test; ^‡^ Mann–Whitney test; ^§^ Fisher’s exact test

With regard to maintaining the same job tasks, there was a trend of patients with a shorter time from trauma to definitive surgery (*p* = 0.069) and sedentary work (*p* = 0.072) to be more likely to maintain the same tasks. None of the remaining characteristics was associated with maintenance of the same job tasks upon job resumption.

The univariate regression models investigating the relationship of patient characteristics with leaves of absence are shown in Table 3. We found highly significant evidence that polytrauma patients have more than twice as many days off work (representing an increase of 120 % of the average days of work absence; *p* < 0.0001) compared with non-polytrauma patients. In addition, there was evidence for an association of time to get back to work with both the time elapsing between trauma and definitive surgery and with ICU admission. The average increase in days off work was 41.3 % (*p* = 0.018) for each additional 10 days from trauma to definitive surgery and 62.9 % (*p* = 0.019) for patients who had been admitted to ICU compared to patients with no ICU admission. None of the remaining study variables were significantly related to leaves of absence.

**Table 3:** Univariate linear regression models evaluating the relationship between leave of absence (in days) and clinical and job-related characteristics of the study participants: percent change, 95 % confidence intervals (CIs), and *P* values

Variables	Category/increment	Percent change (95 % CI)	*P* value
Gender	Female	Baseline	0.154
	Male	27.2 (−53.3, 13.2)	
Age at surgery	10 years more	−0.2 (−15.2, 17.4)	0.979
Time from trauma to definitive surgery	10 days more	41.3 (6.5, 87.4)	0.018
Type of fracture	B	Baseline	0.995
	C	0.2 (−38.7, 63.6)	
Polytrauma	No	Baseline	<0.0001
	Yes	120.1 (45.9, 232.3)	
ASA classification score	0–1	Baseline	0.997
	2–3	0.1 (−42.7, 74.9)	
ICU admission	No	Baseline	0.019
	Yes	62.9 (9.0, 143.5)	
Sedentary worker	No	Baseline	0.620
	Yes	11.3 (−27.7, 71.3)	
Job sector	Private	Baseline	0.096
	Public	51.0 (−7.4, 146.2)	

The models predicted log_10_-transformed leaves of absence. All parameter estimates have been exponentiated

*ASA* American Society of Anesthesiologists physical status classification; *ICU* intensive care unit

## Discussion

Pelvic fractures severely affect the post-trauma work productivity of the patient. In this study, we evaluated how factors, such as complexity of the fracture, pre-trauma health status, time from trauma to definitive surgery, severity of injury, and job characteristics influenced patients’ return to work, the ability to maintain the former job tasks, and the extent of leave of absence.

Many studies [3–18] have described the productivity loss subsequent to these fractures but time to return to work has not been described. We found that the mean time to return to work is approximately 195 days in patients who underwent surgery for a pelvic ring injury.

A systematic review [1] showed work return rates ranging from 57 to 84 % for operatively treated, unstable, and open book pelvic fractures; our population showed similar results (24 % chance of losing the job). Furthermore, our study showed that only 46 % of patients who resumed their job were able to maintain their job tasks, which emphasizes the risk of underestimating the true impact (e.g., through the need for retraining or because of productivity loss) if the ability to return to work is used as the sole outcome.

Our study has several limitations. Patients in an unstable clinical condition could not be transferred to our hospital and were treated by our team of surgeons on-site. They were not included in the study due to the difficulties of retrieving their charts from a hospital other than ours. This ultimately means that our study population is subject to selection bias because the most severe cases are not contained. It is conceivable that the work resumption rates of these patients would probably range at the lower end. Therefore, our results may underestimate the true impact of pelvic fracture on societal costs.

Limitations of this analysis also include selection and information biases as well as general limitations of working with phone interview data. With regard to limitations of the study design, the retrospective collection of data and patient interview limit the conclusions drawn from this study. Furthermore, in our study, only employees from a single European country were included; thus, findings may not be generalizable to other geographical regions.

Our results show also similarity to Gabbe et al.’s study [23]; severity of injury (evaluated by ISS, ICU admission, or both) and not fracture type was identified as an important predictor for work return in both studies. Additionally, we found that the time elapsed between trauma and definitive surgery had a significant correlation with time to return to work, although this parameter may be interpreted as linked to the severity of injury. Time from trauma to definitive surgery has been considered a good indicator of clinical outcomes for pelvic fracture [24]; according to our results, it can also be used to predict productivity loss and, thus, further related costs.

Surprisingly, it appears that work return and leaves of absence are not merely a signal of general decline in pre-trauma health status but that the sudden exogenous trauma generates productivity loss independently of pre-trauma health conditions.

Based on our study, we found that work characteristics (i.e., private or public and sedentary or physically active employment) do not influence work resumption, the ability to maintain the same job tasks, or the number of days of sick leave. However, further studies with larger cohorts are needed to confirm these findings.

Our data did not show correlations between the variables analyzed and return to the same job tasks; we think that the complexity of job types may act as a relevant confounding factor.

Work reintegration after pelvic fracture is a major issue for the patient, health care facilities, and social systems: 58 % of patients were not able to return to work or lost their jobs. Factors correlated with leaves of absence were injury severity, ICU admission, and the time elapsed between trauma and definitive fixation.
